# Causes of Failure in the Use and Application of Porcelain

**Published:** 1908-05-15

**Authors:** Chalmers J. Lyons

**Affiliations:** Jackson, Mich.


### CAUSES OF FAILURE IN THE USE AND APPLICATION OF PORCELAIN.

BY CHALMERS J. LYONS, D. D. S., JACKSON, MICH.

Read before the Port Huron Seventh District Dental Society, April, 1908.

Since the advent of the present century the dental journals have been teeming with their articles on porcelain, until today it indeed seems that there is nothing left to be said. Considerable time has been spent in the discussion of the merits of the high and low fusing porcelain bodies. A great deal of time has also been taken up in discussing the color problems of porcelain, and much time and space has been given to cavity preparation.

Many men have become so enthused over the possibilities of restoring lost natural tooth structure with a material resembling in appearance the tooth structure itself, that they could write or think or talk of nothing but porcelain.

To these over-enthusiastic men we undoubtedly owe much, for their enthusiasm has stimulated the more conservative men to greater activity.

Others, the over-conservative, have condemned the use of porcelain without even investigating it.

To the man between these extremes we owe a debt of gratitude for placing porcelain on the basis on which it now stands, viz: that of one of the *many* useful materials we have today for the purpose of restoring natural tooth structure lost through the ravages of decay. So at this time we can say that gold, the plastics and porcelain all occupy their places in the realm of dentistry, and each, when judicially and conservatively used, has a field which none of the others can fill.

Many good men took up the problem of porcelain a few short years ago, who were careful, painstaking operators, but after a few failures condemned it without trying to ascertain the causes of failure, and today their gasoline and electric furnaces are sweetly reposing in some dusty corner of their laboratories.

So, tonight I want to say something on the "Causes of Failure in the use and application of Porcelain."

I wish to classify the failures under four heads—a failure in any one of them foretelling a complete failure of the whole.

I. Failure in understanding the composition and characteristics of the porcelain body.

II. Failure in cavity preparation.

III. Failure in color combining.

IV. Failure in judgment in deciding where porcelain is indicated and contra indicated.

Under the first head of failure is understanding its composition and characteristics, I want to give you the definition of dental porcelain.

It is defined as a material composed of silicon oxide, the silicates of aluminum, potassium and sodium, which becomes a hard and dense mass by the process of fusion. Now, what do we understand by the term "fusion?" Fusion is a chemico physical change produced by subjecting porcelain to enough heat units for a given time to cause re-arrangement of the molecules with partial vitrification and a glaze of the surface of the mass. This hard, dense mass which is essential in the application of dental porcelain is not procured unless the performance of the operation of fusing is successful.

Dr. John Q. Byram in the *Items of Interest* says: "The baking or fusing of the porcelain body is one of the most important factors in porcelain work, as the results as a whole depend upon its successful performance.

Density, strength, shade and surface gloss are effected by and dependent on the proper conduct of fusing.

Too rapid heating or over-fusing will effect the density and strength by causing perosity and brittleness, and insufficient fusing will impair its crushing strength and gloss.

The given shade of any porcelain depends on its fusing at exactly the heat intended for that special preparation.

The glaze is defective if insufficient heat has been applied, and a glass-like appearance is imparted to the surface by over-fusing.''

When the number of degrees of heat required to fuse the different porcelain bodies was published and adopted by the profession only the half had been told. The time that a porcelain body is subjected to a given degree of heat or a given number of heat units is the important factor in the process of fusion.

It has been clearly demonstrated that porcelain has no definite fusing point; that we can fuse even the consolidated body which is estimated to have a fusing point of 2600 to 2700 degrees heat, on pure gold whose fusing point is 2016 degrees, by subjecting it to a low temperature for a long time.

The lower the temperature and the longer the time given in the process of fusing, the more homogeneous will be the mass, and the characteristic color will be retained.

In not understanding the characteristics of porcelain, shrinkage and the essential requirement of cleanliness have not been given the attention and thought that this work demands. While shrinkage cannot be overcome on account of the composition of the material, it can be controlled so that the operator may definitely know how much shrinkage to prepare for. This can be accomplished by having each mixture of a uniform consistency, mixing it just as thick and dry as it is possible to manipulate it, and by a very thorough condensation drive to the surface and absorb surplus water until the piece is hard and firm. By so doing the crown or inlay will take on the minimum amount of shrinkage in fusing, and it will be more homogeneous in texture.

Absolute cleanliness is another important point in avoiding-failures in porcelain work. I have yet to find a place in dentistry where uncleanliness can be tolerated. Porcelain work is no exception, and perhaps it can be less tolerated in this work than in any other.

The porcelain work should be done on a bench or cabinet, entirely separated from the other work of the office, that it may be scrupulously clean at all times. To be successful, no dirt or oil of any kind or amount should come in contact with the porcelain body or the platinum upon which the porcelain body is to be placed for fusing.

After grinding a crown or inlay and before adding more porcelain body, it should be thoroughly scrubbed with soap and water to remove any foreign matter that may have accumulated from the engine stones. Uncleanliness is usually responsible for failure in procuring dentistry, as any foreign matter in the porcelain body or on the platinum will be turned into gases and these gases in trying to escape will cause porosity in the crown or inlay.

In summing up the first cause of failure: If the cavity preparation has been all right, and the color of the crown or inlay is good, but the piece is lacking in density and strength, then the crown or inlay has been a failure.

Second cause of failure: That of faulty cavity preparation.

No doubt but what this has been the cause of more failures than any of the other causes I have mentioned. Disregarding the foundation work or principles of cavity preparation for inlays, many dentists have become discouraged because they began to construct inlays before they had studied these principles.

The principles of retention of the different fillings, gold, porcelain, etc., must be understood for cavity preparation, for inlays is as distinct as that for gold fillings, and the one must not be confused with the other.

The preparation of many cavities for inlays requires the sacrifice of sound tooth structure in order to secure the retentive resistance that is essential, and to obtain proper color effect. The theory that close adaptation and cement will retain an inlay is a beautiful theory, but the successful dentist cannot build and retain his practice on theory. This theory is adverse to all known laws of mechanics, and will not work out practically in porcelain inlay work. In other words, saucer-shaped cavities without any retention other than the film of cement used in setting the inlay are responsible in the large majority of cases for the failure and subsequent condemnation of this material and process. The subject of cavity preparation for inlays is too broad for the time allotted to me tonight to take it up in all of its details so I will only take the time to mention a few of the essential requirements.

1st. The force and direction of occlusion must be taken into consideration before beginning the preparation of the cavity, then by grooves and angles, secure all the mechanical retention that is possible without forming undercuts.

2nd. The walls of the cavity should slightly diverge towards the margins.

3rd. The cavity should be as deep as conditions will permit with the pulpal wall parallel to the plane of the surface on which the cavity is located.

4th. All undercuts must be obliterated so as to be able to withdraw the matrix without distorting it.

5th. Frictional retention must be secured by having the pulpal wall as extensive as possible without forming undercuts.

6th. Sufficient working space must be secured before beginning the operation.

If these essential laws are not carried out nothing can be expected except failure. We cannot slight or overlook a single one of these principles of cavity preparation and succeed in inlay work.

The third cause of failure is that of not understanding how to combine the different hues of color in order to bring out the proper tone.

When we consider that we are endeavoring to imitate a substance in color that is partly organic matter and partly inorganic matter with a substance wholly inorganic in composition, differing in density and colored with different pigments, we can readily understand how difficult it is.

In order to understand the color problems in porcelain, light, which is the source of color, must be taken into consideration, and the laws of reflection, refraction, diffusion and absorption thoroughly understood.

As light enters the porcelain part of it is reflected, part refracted, part diffused and the remainder absorbed.

The angle of reflection and the amount of reflected light given off depends upon the contour and the surface gloss, while the angle of refraction and the amount of refracted light which enters the porcelain depends upon the density and texture of the porcelain. Here again we see the importance of fusion.

Unless we obtain exactly the same gloss on the surface of the porcelain as that of the natural tooth, the amount of reflected light will differ from that of the natural tooth.

Unless the contour of the crown or inlay is exactly the same as that of the natural tooth, then the angle of reflection will differ, and the appearance will be changed.

The amount of refracted light must necessarily vary on account of the density of the porcelain differing from the density of the natural tooth, but the nearer the density of the natural tooth structure can be approached, the more nearly will the refraction be equalized.

The normal dentine under a high power glass resembles fine sand paper in appearance, and in building up the porcelain body that is to represent the dentine, the nearer we can approach that dull appearance, the nearer we can approach the appearance of the natural tooth when our work is completed. By not over-fusing the foundation body at any time in the process of constructing a crown or inlay, that dull appearance will be left, so that instead of all of the light being reflected a part of it will be diffused, and the conditions will more closely resemble the conditions of nature.

The remaining property of light to contend with is absorption, and this is one of the hardest to overcome.

Any foreign substance placed back of a translucent body will form a dark background, and light will be absorbed instead of diffused, unless that translucent body is thick enough so that the rays of light will be reflected, refracted and diffused before reaching that body.

The thicker and deeper the inlay, the less absorption of light will take place. The cement should be as light as possible, as less absorption occurs when there is least pigment in the cement.

In building on the enamel colors I have been more successful in building them on by sections rather than in layers, because the hues in the underlying layers will affect the color in the over-lying layers. For instance, if we attempt to build a yellow over a blue, we do not get a darker yellow or lighter blue, but a green is produced.

To be successful in building up in layers it is necessary to understand thoroughly all color combinations, that is, what effect each color and thickness of that color will have over those layers underlying, and to know just what hue and amount of that hue is necessary to produce the desired results in matching the natural tooth.

In building up in sections I use the foundation body to represent the dentine, usually using a yellow, then build in the gingival third with the color desired, then the middle third, then the incisal third, not having these colors overlap, and only fired to a hard biscuit, finishing the whole by a uniform layer of neutral color to obtain the surface gloss.

In matching a tooth for crown or inlay, experience, judgment and deliberation should be exercised; then when the different hues have been decided upon to bring about the proper tone of the color of the natural tooth, care in fusing should be exercised, so that those hues will be developed successfully.

The aesthetic value is the chief factor in the use and application of porcelain, and if we have overcome all of the other causes of failure and in the end have not obtained the required color, then is the whole operation a failure.

The fourth cause of failure in porcelain work that I have mentioned is, failure in judgment as to where porcelain is indicated and contra indicated. Next to cavity preparation, this has no doubt led to more failures than anything else.

It is unfortunate that the dental profession as a whole are too eager to take up and expand the new ideas without first giving to them the thought that many of these ideas merit. It seems that every new material or process that is given to the dental profession is eagerly grasped. Men become enthusiastic over and advocate them far and wide before the merits or demerits are ascertained. These new ideas many times get into the hands of men who are unable to bring out the best that is in the material or process, and much harm is consequently done the profession and also the public.

In the cataphoric days the profession thought that pain attendant upon a dental operation was a thing of the past, but the ''painless dentists'' are not nearly so numerous today as they were six or seven years ago. The method was all right, and is all right in some cases, and some of the appliances were all right, but the fault was with the men. It was abused before it was understood.

Today the profession has become excited over the gold inlay. I feel that there has been nothing given the profession in the last decade that is as valuable when conservatively used as the cast gold inlay. But today it is not used conservatively. Many men think it has no limitations, and are placing it in every cavity that they can, large, small and medium, and it will take some time for it to get down on a solid basis as one of the valuable adjuncts of dentistry. The silicate cements are likewise going through their stages of development, and are being used indiscriminately the same as porcelain was, without reference or thought as to the places where they are indicated, and with little knowledge as to the characteristics of the material.

In the beginning of the porcelain craze many men supposed that the culmination of dental art had been achieved. Some men made the statement that porcelain was limited as a filling material only by the limitations of the operators' skill; that it was indicated in any and every place where gold was. As time went on, more became known about the composition and characteristics of porcelain body, and today with the conservative, conscientious man it merely takes its place beside the many other materials that have served us long and well as a material that has a useful field in dentistry.

Porcelain is not, nor has it ever been indicated where undue strength is demanded either in crown or inlay work. We have to depend upon bulk of porcelain for strength in this material, and it is never indicated where there is not room enough between the opposing teeth for a sufficient bulk of porcelain to impart the required strength to the piece necessary to withstand the force of occlusion in mastication. For this reason, I have always believed that gold was a much better material in the bicuspids and molars for inlays, and also much better in the molars for crowns than porcelain.

In closing, let me say that porcelain is one of the most valuable materials we have in the realm of dentistry today when judiciously and conservatively used. It is a material that to be successful in its application every detail in its use and manipulation must be thoroughly accomplished, and more than this with all of the fine manipulative ability required in the successful performance of an operation in porcelain, good judgment and common sense should always prevail in its application.

